# Social Participation of Diabetes and Ex-Leprosy Patients in the Netherlands and Patient Preference for Combined Self-Care Groups

**DOI:** 10.3389/fmed.2014.00021

**Published:** 2014-08-18

**Authors:** Henry J. C. de Vries, Roos de Groot, Wim H. van Brakel

**Affiliations:** ^1^Department of Dermatology, Academic Medical Centre University of Amsterdam, Amsterdam, Netherlands; ^2^STI Outpatient Clinic, Cluster for Infectious Diseases, Municipal Health Service (GGD), Amsterdam, Netherlands; ^3^Centre for Immunity and Infectious Diseases Amsterdam (CINIMA), Academic Medical Centre, University of Amsterdam, Amsterdam, Netherlands; ^4^Netherlands Leprosy Relief (NLR), Amsterdam, Netherlands

**Keywords:** leprosy, diabetes, stigma, self-care groups, neuropathy, social participation

## Abstract

**Introduction:** Earlier, we showed that neuropathic complications limit social participation of ex-leprosy patients, even in a non-endemic leprosy setting like the Netherlands. Self-care groups for ex-leprosy patients can strengthen self-worth of participants, prevent further handicap, and enable the exchange of coping strategies. For non-endemic leprosy settings with a very low rate of leprosy patients, a self-care group exclusively for (ex)leprosy patients is not likely to be feasible. A combined group with patients facing comparable morbidity would be more efficient than disease-specific self-care groups. Here, we studied the comparability in social constraints of diabetic patients and ex-leprosy patients. Moreover, we investigated if combined self-care groups for ex-leprosy patients and diabetic patients would be desirable and acceptable for possible participants.

**Methods:** Social participation was studied based on in-depth interviews and Participation Scale information collected from 41 diabetic patients and compared with the data of 31 ex-leprosy patients from a prior study. Moreover, we made an inventory of potential strengths and limitations and attitudes toward combined self-care groups for diabetic patients with neuropathy.

**Results:** The following themes emerged among diabetic patients: disease confrontation, dependency, conflict with partner or relatives, feelings of inferiority, stigma, abandoning social activities, fear of the future, lack of information, and hiding the disease. These themes were very similar to those voiced by the previously interviewed ex-leprosy patients. The latter more often mentioned stigma and disease ignorance among Dutch health care workers. Whereas ex-leprosy patients perceived stigma on multiple fronts, diabetic patients only mentioned feeling inferior. Diabetic patients experienced some form of participation restriction in 39% of the cases as opposed to 71% of the ex-leprosy patients. Diabetic patients did acknowledge the comparability with leprosy as far as their neuropathic complaints concerned. Yet only 17% showed interest in combined self-care groups. The majority preferred disease-specific self-care groups only focused on diabetic patients. This might have been caused partly by the perception that a self-care group is yet another disease-related demand on their time, rather than an opportunity to become less dependent on health care services.

**Conclusion:** The physical complications and social problems in ex-leprosy and diabetic patients with neuropathy are similar. Both groups show social participation limitations, yet in contrast to diabetic patients, ex-leprosy patients perceive stigma in more domains in life. Despite the fact that diabetic patients preferred disease-specific, homogeneous self-care groups, we believe that the option of combined groups with ex-leprosy patients and possibly even other people needing chronic wound care is a promising strategy. Therefore, further research is warranted into the acceptance and impact of self-care groups as a strategy to reduce social constraints by diseases causing neuropathy.

## Introduction

A disease that requires chronic care can affect relationships and friendships and can cause limitations in the social and civic functioning of those affected (1, 2). Self-managing one’s own condition can help patients to tackle some of these day-to-day problems. The integration of self-management into health care in the form of self-care groups can support patients with chronic conditions and enhance social participation and self-esteem (3). Self-care groups consist of persons living with a similar chronic condition and/or disability that meet on a regular basis for support, to share problems and exchange coping strategies (4). The goal of these groups is to increase participants’ empowerment, self-reliance, and coping with the chronic condition or disability (5, 6). Self-care groups for cancer patients has shown to increase the social participation through portraying positive role models (7). Self-care groups for leprosy patients have been evaluated in Ethiopia (8) and in Nepal (9), with positive outcomes. The Ethiopian study showed a decrease in the occurrence of neuropathic ulcers, whereas the Nepalese study showed that participation in a self-care group improved social participation and self-respect.

Earlier, we showed that neuropathic complications limit social participation of ex-leprosy patients, even in a non-endemic leprosy setting like the Netherlands (10). A majority of the interviewed patients avoided social activities and were afraid that neuropathic complications would occur or be aggravated through daily activities. Moreover, (self) stigma was a major cause of social problems. This was surprising since the concept of leprosy has vanished in the general population in the Netherlands. It was concluded that, despite the large burden of disease for the individuals concerned, there is no specific attention to the social problems of ex-leprosy patients in the Netherlands, and that they could benefit from programs that increase the social interaction between peers, like self-care groups.

Since there are relatively few ex-leprosy patients living in the Netherlands, it is difficult to organize disease-specific self-care groups. It has previously been suggested that combined self-care groups for (ex) leprosy patients and people with other conditions that cause similar disease-related problems (for example, diabetic patients with neuropathic complications) could overcome the problem in low and non-endemic countries of small numbers of potential participants living too far apart to form groups effectively (11). Diabetes is the most prevalent chronic condition in the Netherlands. Several studies have shown the socially limiting effect of diabetes (1, 12).

In this study, we examined the impact of diabetes on the social functioning of patients, in domains such as family life, work, relationships, and social contacts, plus the impact of social functioning on self-management of diabetes. We then compared the findings with data derived from an earlier study with a similar design on the social problems among ex-leprosy patients in the Netherlands in which the same research instruments were applied (10). The research questions were: (1) To what extent are the social participation restrictions caused by neuropathy among ex-leprosy and diabetic patients comparable? (2) Are diabetic patients interested in combined self-care groups for both ex-leprosy and diabetic patients?

## Materials and Methods

### Design

This is an observational, descriptive cross-sectional study with a focus on the patients’ view.

### Study sample

Diabetic patients (both insulin and non-insulin-dependent) with neuropathic complications were approached at three different health care centers in the Netherlands: (1) a university hospital (Academic Medical Centre, University of Amsterdam), a general hospital (Reinier de Graaf Hospital, Delft), and a primary health care centre (De Roerdomp, Nieuwegein). The criteria for inclusion were having diabetes with neuropathy and willingness to give verbal informed consent.

### Data collection and instruments used

The physician responsible for treatment approached patients about participation in the study. Upon consent, the interviewer (R. de Groot) met the participants for an interview session of approximately 45 min at their own home or in the hospital. All interviews were conducted in Dutch and were audio-recorded.

### Analysis

To compare the group of diabetes and ex-leprosy patients, the same questionnaire, Participation Scale, and interview guide were used as described before (10). In short, qualitative data was obtained with validated semi-structured interviews (13). The following items were included in the interview-guideline: basic knowledge on diabetes, social participation, self-care, discrimination, social relationships, exclusion, restrictions, and self-esteem. The questionnaire used in this study was an adapted version of a validated interview guide (14).

Quantitative data was derived from the Participation Scale, an instrument based on the terminology and conceptual framework of the International Classification of Functioning, Disability, and Health (ICF) (15). This scale was used to measure the impact of stigma (16, 17). It has been validated for use with people affected by chronic conditions and for use in the Netherlands (16). The scale contains 18 questions on experienced participation restriction and interviewees are asked to compare themselves with persons who are not affected by diabetes. The Participation Scale has a total score on a scale from 0 to 90; a score of less than 13 means no significant participation restriction, 13–22 equals mild restriction, 23–33 moderate restriction, 34–53 severe restriction, and more than 53 is classified as extreme restriction (16). Diagnostic delay was defined as “a point of consultation along the referral pathway where no diagnosis or misdiagnosis is made (18).

### Ethical considerations

The ethical committee of the Academic Medical Centre of the University of Amsterdam issued a statement of “no objection” against the study. A proof in writing of this statement is in possession of the authors. All eligible subjects were asked for verbal informed consent prior to the interview. No incentives were paid.

## Results

Forty-one diabetic patients with neuropathy were interviewed with an average age of 67 years. The group consists of 27 men and 14 women of which 33 people of Dutch descent and 8 of Surinamese descent. Sixteen patients have been enrolled at the University Hospital, 19 at the general hospital, and 6 at the primary healthcare centre. Three patients had type 1 and 38 type 2 diabetes. Table 1 provides the characteristics of both the diabetic patients and the ex-leprosy patient group.

**Table 1:** Characteristics of 41 neuropathy patients with diabetes and 31 ex-leprosy patients*, The Netherlands, 2010–2012.

Characteristic	Diabetic patients	Ex-leprosy patients
Average age (years)	67	58
Sex (males/females)	27/14	12/19
Origin (country of birth)		
The Netherlands	33	0
Suriname	8	17
Other	0	14
Number under care for condition	41	21

*The ex-leprosy patients were described previously in Ref. (10).

### Daily confrontation with the condition

The diabetic patients were confronted with sometimes confusing demands related to their condition, especially, the drug regimen and fixed meal times. Although ex-leprosy patients are not confronted by strict medication or dietary rules, 16 out of 41 (39%) also mentioned having to deal with their illness or its consequences on a daily basis. Especially, the prevention of neuropathic ulcers by wearing orthopedic shoes and the inspection and bandaging of ulcers was mentioned as reoccurring daily burden. Both patient groups mentioned difficulties in controlling the course of their illness; they felt frustration when complications like neuropathic ulceration occurred or worsened. This is clearly illustrated by two participant quotes: “*I try as much not to think of how my feet could get worse, in the end there is little you can do about it*.” and “*It is praying, hoping and begging that my foot does not deteriorate further*.”

### Dependency

Both patients groups mention dependence with regard to mobility as a major problem affecting their self-esteem. Like ex-leprosy patients, diabetic patients found it very difficult having to rely on assistance while traveling. “*As a cause of my diabetes I am very dependent on my husband. He always has to escort me while I sometimes would like to shop alone*.”

### Conflict with partner or relatives

Nine out of 41 participants (22%) mentioned the inability to perform joint activities with partner or relatives. They had to abandon shared hobbies, outdoor activities like cycling, or spending holidays abroad together, out of fear of having to be hospitalized in a foreign hospital. In addition, sexual problems caused by impotence and/or libido loss were mentioned as a cause of stress within relationships. Likewise, ex-leprosy patients mentioned conflicts with partner or relatives. In their case, the source of conflicts was mainly the patients’ wish to conceal the illness that had negatively affected the relationship with their partner or relatives.

### Feelings of inferiority

Nine of the 41 (22%) diabetic patients felt less worthy and had a low self-esteem. This was often related to unemployment and an inability to participate in society, and sometimes caused feelings of depression. A 54-year-old women with diabetes said: “*If I did not have a child I would lie in bed all day. I panic at the idea that I am home-bound and so very dependent on others*.” Likewise, 12 out of the 31 (38.7%) ex-leprosy patients also had a low self-esteem. They felt “different” and “weird” compared to people without leprosy.

### Stigma

A remarkable difference between ex-leprosy and diabetic patients was the degree of perceived stigma. A considerable proportion of the diabetic patients (34%) did not often discuss their illness with their partner or relatives, mostly out of fear to burden them with their problems. An even larger portion of the ex-leprosy patients kept their illness a secret for their partner or relatives mainly due to embarrassment. Twenty-five out of the 31 (81%) ex-leprosy patients suffered from negative associations and feelings of shame concerning their condition, which is considered self-stigma. In contrast, none of the diabetic patients experienced direct stigma; almost all spoke openly about diabetes. Mr. G, 66 years old, said: “*Why should I be embarrassed? It’s something everyone can get*.”

### Fear of the future

Like with ex-leprosy patients, the interviewed diabetic patients are deterred by terrifying disease-associated stories of peers. As a consequence, 6 of 41 would rather not think about future. Mrs. O (54): “*I’ve seen other diabetic patients when I was in the hospital. It all started with the amputation of a toe and within one and half years they got amputated up to their knees*.”

### Disease insight and diagnostic delay

Whereas only 15% of the diabetic patients showed poor or no knowledge of their disease diabetes, 65% of the ex-leprosy patients indicated they lack good information about their disease. In contrast to ex-leprosy patients who experienced a diagnostic delay of 1.7 years, none of the diabetic patients mentioned delay. All were either diagnosed during a routine check, or immediately after they consulted their general practitioner because of diabetes-specific symptoms. Ex-leprosy patients mentioned experiences with doctors and specialists that had no clue about the nature of their complaints and symptoms.

### Participation scores

The median participation score of diabetic patients was 6.0 (IQR 16.5), which represents no significant participation restriction. Among the diabetic patients, 16/41 (39%) experienced some form of participation restriction (a score of 12 or higher). In contrast, ex-leprosy patients had a median participation score of 16 (IQR 33), which equals mild restriction, and 22/31 (71%) reported some form of participation restriction. The most common moderate or major perceived participation restriction among 15/41 (37%) diabetic patients was the possibility to maintain a relationship, in contrast to 3/31 (9.7%) among ex-leprosy patients (Table 2). Second, participation in social activities was perceived as a major problem by 13/41 (32%) of the diabetic patients, which is similar to the proportion found in ex-leprosy patients (36%). Much can be attributed to mobility-related restrictions; Mrs J (54) says: “*I cannot walk far, after 100 meters I have a lot of pain. That’s why I stay over at home*.” As a consequence patients lack social contacts.

**Table 2:** Percentages of 41 neuropathy patients with diabetes and 31 ex-leprosy patients* with moderate to large restriction scores on 18 participation items^#^, the Netherlands, 2010–2012.

Item	Diabetic patients (%)	Ex-leprosy patients (%)
Maintain relationships	36.6	9.7
Participate in social activities	31.7	35.5
Perform same amount of work	19.5	29.0
Participate in events	19.5	22.6
Visit people in proximity	19.5	25.8
Travel to locations outside residence	17.1	32.3
Find employment	17.1	29.0
Contribute to family income	17.1	19.4
Learn new things	14.6	22.6
Move in/around house	12.2	22.6
Take care of yourself	9.8	29.0
Perform household work	9.8	19.4
Visit public places	7.3	25.8
Active citizenship	4.9	12.9
Being approached with respect	0.0	22.6
Express one’s opinion	0.0	16.1
Help others	0.0	6.5
Approach strangers with confidence	0.0	32.3

*The ex-leprosy patients were described previously in Ref. (10).

*^#^Based on the terminology and conceptual framework of the Participation Scale of the International Classification of Functioning, Disability and Health (ICF), available at: http://www.rivm.nl/who-fic/icf.htm*.

### Self-care groups

Only 7 of the 41 diabetic patients (17%) responded with enthusiasm to the concept of self-care groups for patients with neuropathic problems (caused by leprosy or diabetes), with an emphasis on the exchange of practical information, tips, and incidental social interaction. Although some felt a need to exchange experiences, the majority stated that they already spent a lot of time on hospital visits and engagement in self-care groups would increase the already perceived preoccupation with their illness.

After additional explanation, most participants understood the shared burden with ex-leprosy patients. Although 17% of the diabetic patients thought that combined self-care groups with ex-leprosy patients could succeed, all stated a preference for self-care groups specifically for patients with diabetic neuropathy. Besides being with peers, they would prefer group members with a comparable level of education. As a 66-year-old male participant stated: “*I’d like to participate in a self-care group, but only with people who have some idea of what I’m going through. Even then, it would be hard to compare because every person experiences his or her diabetes differently*.”

## Discussion

The main outcome of this study is that diabetic patients with neuropathic complications experience restrictions in their social participation, comparable to the limitations found among ex-leprosy patients in our recent survey (10). Although the median participation score of diabetic patients is lower than that of the ex-leprosy patients, the proportion that experience social participation problems is comparable in several ways. A considerable proportion of both ex-leprosy patients and diabetic patients experience a moderate to severe restriction in their daily lives to participate in social activities and special events, the ability to find employment, the ability to socialize, and in their mobility.

Although ex-leprosy patients suffer from feelings of shame, secretiveness, and fear of disclosure much more than diabetic patients, both groups are confronted by many similar day-to-day limitations as a consequence of their neuropathic complications. Both groups mention being less mobile, more dependent on others, and thus being home-bound. Also the feeling of having little grip on disease-related complications, and forced abandonment of hobbies, work, or sports is common both among diabetic (2) and ex-leprosy patients.

As far as the second research question is concerned (are diabetic patients interested in combined self-care groups for both ex-leprosy and diabetic patients?), the outcome of this study was negative. From our previous study, we learned that ex-leprosy patients have a need for interaction with other patients, for example in the form of self-care groups. A complaint of many ex-leprosy patients in the Netherlands is the lack of information about their disease. Many healthcare providers are not well informed, let alone the society at large. One of the striking quotes from an ex-leprosy patient was: “*You never read or hear anything about leprosy in the Netherlands, it is like it does not exist*” (10). The public ignorance concerning their illness experienced by ex-leprosy patients is not experienced by diabetic patients. Diabetes is a much prevalent illness and the general public is well aware of diabetes.

Diabetic patients seemed to experience little illness-related stigma; they are not ashamed of their disease, do not experience negative reactions from society and do not mention being treated differently because of diabetes. Nonetheless, quite a few patients with diabetes (22%) mentioned feeling less worthy or having a low self-esteem, which is a form of internalized-stigma. Several studies have shown that diabetic patients are sometimes considered lazy, immoderate, and a burden on their family and relatives (19, 20). This is probably caused by the relation between insulin-dependent diabetes and obesity. Other forms of stigma have been reported, like negative reactions when injecting insulin (21), employers disadvantaging diabetic patients for their condition (21), and patients hiding their illness out of fear of negative treatment (22). We attribute the minimally reported stigma in our survey to the relative awareness of the disease in the Netherlands.

Research has shown that self-care groups for diabetic patients can improve their psychosocial and physical outcomes (23, 24). In the Netherlands, self-care groups for diabetic patients are in development with a focus on cognitive behavioral therapy (25, 26). We envisaged combined self-care groups for ex-leprosy patients and diabetic patients that focus on social participation and the prevention and care of neuropathic complications. Instead of assembling participants into groups based on a shared illness, these groups would have disease-related complications as a binding factor. This study shows that most diabetic patients say they do not want to participate in a self-care group. There are several reasons for this. First, they believe these meetings will be time consuming and would come on top of the already burdensome necessary routine visits to various health services. Evidence shows that practicing self-care, particularly in groups, can reduce the prevalence and incidence of wounds and reduce the risk of repeat admission for chronic wounds among persons affected by leprosy (27, 28). Apart from empowerment and social interaction, self-care groups are especially meant to prevent and heighten alertness on the early signs of neuropathic complications. As a result, joining a self-care group could reduce the need for additional care and the time spent in routine health care settings. It is therefore important to communicate the expected benefits form self-care groups to patients. Second, they fear to be confronted with negative disease-related stories of group members. In a small group setting, this would be much less likely than e.g., during hospital admission.

Of those participants that did feel they could benefit from self-care groups, the majority did not see added value in the participation of ex-leprosy patients. Although they acknowledge the rationale of combined groups, they preferred disease homogenous groups focused on diabetic patients only. Moreover, similar social and educational background within self-care groups is also mentioned as a key to successful meetings. Unpublished experience from a pilot project on joint diabetes-leprosy self-care groups in Indonesia suggests that there is sufficient common ground between the two groups and that social differences and stigma does not hinder group function as had previously been feared. As mentioned earlier, self-care groups focused on ex-leprosy patients only seem not a viable option in non-endemic settings like in the Netherlands or in other low-endemic settings. Apart from the restricted mobility of those involved, patients tend to live too far apart to form viable long lasting groups that can meet on a regular basis. As an alternative platform to fulfill the need for social interaction with peers, social media, and web-based initiatives could be evaluated.

A recent study among 227 professionals in the field of diabetes and/or leprosy showed that they acknowledged the similarities between the two conditions, both physically and psychosocially (3). Considerable overlap in current practices was found, mainly in patient education in prevention of disability, skin assessment, and skin care, and the recommendation to use protective footwear. The majority of respondents considered combined self-management interventions to be “possible” (33.3%) or “possible and promising” (30.8%).

A weakness of this study is the different periods in which the ex-leprosy and diabetic patients were interviewed. Since the same interviewer performed the data collection and the same analysis methods were used, we still feel that the data can be usefully compared. Moreover, we have no data on the willingness of ex-leprosy patients to participate in combined self-care groups. The ex-leprosy patients were interviewed before we envisaged the possibility of self-care groups. A strength of this study is its extensive qualitative information that can serve as a starting point for future studies on combined self-care groups. Not only in non-endemic settings, but particularly also in leprosy-endemic countries, there is a great need for patient-centered care initiatives that can reduce dependency on the limited resources, empower patients, and increase social participation. Apart from leprosy or diabetes, research is needed on interventions against social isolation of people with diseases that cause chronic need of care.

We conclude that the similarity in social participation restrictions both among ex-leprosy and diabetic patients opens opportunities to offer combined self-care groups for both populations. Despite the fact that diabetic patients said to prefer disease-specific, homogeneous self-care groups, many professionals in the field of diabetes, and leprosy are positive about the prospects of joint self-management interventions. It would therefore be worthwhile to explore combined groups with ex-leprosy patients and perhaps others needing skin care in some form further in future research, both in the Netherlands and in leprosy-endemic settings.

## Author Contributions

Henry J. C. de Vries and Wim H. van Brakel designed the study: Roos de Groot collected the data, Henry J. C. de Vries, Roos de Groot and Wim H. van Brakel interpreted the data, and wrote the draft manuscript. Henry J. C. de Vries approved the final manuscript version to be published.

## Conflict of Interest Statement

The Review Editor Adele Zingoni declares that, despite having collaborated with author Henry J. C. de Vries, the review process was handled objectively and no conflict of interest exists. The authors declare that the research was conducted in the absence of any commercial or financial relationships that could be construed as a potential conflict of interest.
